# A comparison of a novel time-based summary measure of dairy cow health against cumulative disease frequency

**DOI:** 10.1186/s13620-018-0119-z

**Published:** 2018-03-02

**Authors:** Craig S. McConnel, Ashleigh A. McNeil, Joleen C. Hadrich, Jason E. Lombard, Jane Heller, Franklyn B. Garry

**Affiliations:** 1 Veterinary Medicine Extension, Department of Veterinary Clinical Sciences, College of Veterinary Medicine, Washington State University, WA 99164, PO Box 646610, Pullman, WA USA; 2 Integrated Livestock Management, College of Veterinary Medicine and Biomedical Sciences, Colorado State University, Fort Collins, CO 80523 USA; 3 Department of Applied Economics, College of Food, Agricultural and Natural Resource Sciences, University of Minnesota, St. Paul, MN 55108 USA; 4 USDA:APHIS:VS, Centers for Epidemiology and Animal Health, 2150 Centre Ave., Bldg. B, Fort Collins, CO 80526 USA; 5 School of Animal and Veterinary Science, Charles Sturt University, Wagga Wagga, NSW 2650 Australia; 6 Graham Centre for Agricultural Innovation (NSW Department of Primary Industries and Charles Sturt University), Wagga Wagga, NSW 2650 Australia

**Keywords:** Dairy cow, Morbidity, Mortality, Culling, Disease-adjusted, Lactation

## Abstract

**Background:**

There is an increasing push for dairy production to be scientifically grounded and ethically responsible in the oversight of animal health and well-being. Addressing underlying challenges affecting the quality and length of productive life necessitates novel assessment and accountability metrics. Human medical epidemiologists developed the Disability-Adjusted Life Year metric as a summary measure of health addressing the complementary nature of disease and death. The goal of this project was to develop and implement a dairy Disease-Adjusted Lactation (DALact) summary measure of health, as a comparison against cumulative disease frequency.

**Methods:**

A total of 5694 cows were enrolled at freshening from January 1st, 2014 through May 26th, 2015 on 3 similarly managed U.S. Midwestern Plains’ region dairies. Eleven health categories of interest were tracked from enrollment until culling, death, or the study’s completion date. The DALact accounted for the days of life lost due to illness, forced removal, and death relative to the average lactation length across the participating farms.

**Results:**

The DALact consistently identified mastitis as the primary disease of concern on all 3 dairies (19,007–23,955 days lost). Secondary issues included musculoskeletal injuries (19,559 days), pneumonia (11,034 days), or lameness (8858 days). By comparison, cumulative frequency measures pointed to mastitis (31–50%) and lameness (25–54%) as the 2 most frequent diseases. Notably, the DALact provided a robust accounting of health events such as musculoskeletal injuries (5010–19,559 days) and calving trauma (2952–5868 days) otherwise overlooked by frequency measures (0–3%).

**Conclusions:**

The DALact provides a time-based method for assessing the overall burden of disease on dairies. It is important to emphasize that a summary measure of dairy health goes beyond simply linking morbidity to culling and mortality in a standardized fashion. A summary measure speaks to the burden of disease on both the well-being and productivity of individuals and populations. When framed as lost days, years, or lactations the various health issues on a farm are more comprehensible than they may be by frequency measures alone. Such an alternative accounting of disease highlights the lost opportunity costs of production as well as the burden of disease on life as a whole.

## Background

Over the past decades there has been an increasing awareness of the detrimental impact on profitability and welfare due to rising levels of dairy cow mortality in the U.S. and abroad [1–3]. Similarly, the consequences of forced or biological culling of dairy cows due to ill health and injury have raised concerns regarding animal well-being and economic opportunity costs [4, 5]. Underlying these issues are the health and welfare implications of conditions such as lameness and mastitis, which have the potential to decrease production and cause pain and suffering. Such costly diseases or injuries pose an economic problem for farmers and raise the broader question of cow longevity in contemporary production [6].

Standardized accounting of dairy culling and mortality is necessary to appreciate the impact of underlying disease and injury, and the extent to which interventions influence associated risk factors [7]. Dairy record systems have historically focused on non-fatal health problems in an effort to monitor impacts on milk production and drug residues. Information regarding early exit from the herd due to disease or death is often fragmented, inconsistent, and rarely viewed within the context of underlying disease states [8, 9].

For the most part diseases are recorded based on treatments, and analyzed through frequency measures independent of their outcomes. Their impact is typically framed in isolation or in terms of the cost of treatments and lost milk production [10–12]. Even in those studies where the cost of disease incorporates more nuanced accountings such as demands on employees’ time, reduced reproductive performance, and effects on longevity, the costs are related to current prices and production standards making it difficult for comparative analyses across time and location [13–15]. Although such studies are important and useful in providing guidelines for optimizing health care management, it is worth considering alternative methods for standardizing the impact of health problems across time, populations, disease states, and outcomes.

Human medical epidemiology has attempted to standardize the impact of disease through a time-based measure of health. This summary measure is termed a Disability-Adjusted Life Year (DALY) and accounts for both the years of life lost due to premature mortality and the years of life lived in less than ideal health [16, 17]. The combination of non-fatal health outcomes with mortality provides a comprehensive framework to equitably assess both individual and population levels of disease burden [18]. The DALY framework was developed such that health outcomes that represent a loss of welfare are included, similar health outcomes are treated the same to ensure comparability of the burden of disease across different populations or in the same population over time, and time is the unit of measure for the burden of disease [19]. This method for standardizing the impact of disease in humans provides an assessment of the effectiveness of health systems that helps health authorities prioritize actions and allocate resources to reduce preventable disease and death.

Calculating the DALY requires consistent measures of health loss in the form of so-called disability weights [19–22]. Disability weights reflect the relative severity of important diseases using a number on a scale from 0 to 1, with a value of 0 representing perfect health and 1 representing a state equivalent to death. Although there has been extensive debate regarding the definition and measurement of disability weights [20], the latest human disability weights capture the most salient differences in clinical symptoms and functionality [23]. With the help of disability weights, part of the time lived with a disease is regarded as not lived and the remainder is regarded as time lived in good health [21].

Ultimately the DALY accounts for the years of life lost due to disability (YLD) and premature death (YLL). The YLD originally was calculated as a function comprised of *I x DW x L* where *I* refers to the number of incident cases, *DW* the disability weight, and *L* the average duration of a health problem until remission or death [24]. The use of an incidence-based YLD approach aimed to ensure consistency with the YLL calculation, which is inherently incidence-based as well. More recently, the YLD has been calculated using a prevalence-based approach comprised of *DW x p* where *DW* remains the disability weight, and *p* refers to the prevalent cases during a given year. This approach is intended to overcome incidence-based challenges related to measuring the current prevalent burden of disease for which incident cases have decreased, estimating average durations of disease, and assigning diseases to the age-group at which they occur. The major impacts of the prevalence approach include significantly shifting the age distribution of YLD across a lifetime for certain disease states, while imparting greater weight to deaths compared to non-fatal health loss [22, 23]. Regardless of the underlying methodology, the YLD measure is added to the YLL which is calculated as *N* x *L* where *N* represents the number of deaths due to a cause, and *L* is a standard loss function describing years of life lost relative to an expected lifespan [22, 24]. The DALY product consequently describes the overall health loss from a given disease or disease group that can be assessed across time, populations, and regions.

The goal of this project was to develop and implement a dairy disease-adjusted summary measure of health comparable to the DALY, as a comparison against a basic accounting of disease in the form of cumulative frequency (i.e. the sum of unique incidents of disease). Guidelines have been recommended for calculating and reporting disease occurrence [25], but the reality is that many producers do not record diseases in a manner useful for documenting measures such as incidence and severity [26]. Although current dairy health data recording is of variable and generally poor quality, new disease episodes, repeat episodes, and death and culling provide objective measures of health management that should be routinely monitored [27]. This project built upon previous efforts to enhance the documentation of dairy cow death [28], and to better understand the impact of disease on productive life and well-being [29]. It was founded on the presumption that dairies would benefit from *a time-based accounting* of *the burden of disease inclusive of morbidity, culling, and mortality*. Such a consistent and comparative descriptor would provide a platform for ongoing assessments of the impacts of disease, an incentive for improving disease data recording, and ultimately enhance health decision-making and planning processes. The dairy industry is well versed in the effects of ill-health on milk production [30, 31]. Expanding the discussion to view profits and losses in light of the quality and length of productive life would provide dairy health managers alternative insight and perspective.

## Methods

### Dairy records

The data set for this project was derived from approximately 8000 primarily Holstein lactating cows housed in free stall barns on 3 similarly managed commercial dairies in the Midwestern Plains’ region of the U.S. Average percentages of cows within the first, second, and third or greater lactations per farm were as follows: Dairy 1 (32%, 32%, 36%); Dairy 2 (43%, 33%, 24%); Dairy 3 (32%, 27%, 41%). Management included 3 times per day milking and total mixed rations. Cows on the 3 dairies were enrolled at the time of freshening over the course of 17 months extending from January 1st, 2014 through May 26th, 2015. Cows that freshened twice during the study were enrolled a second time. Dairy cow disease, culling, and mortality data were acquired for each enrolled cow from herd data backups using the EVENTS function in the on-farm dairy management (i.e. reproduction, production, and health) computer software program DairyComp 305 (Valley Agricultural Software, Tulare CA, USA, 2015). These data were downloaded on a monthly basis into an Excel spreadsheet (Microsoft, 2013) designed to organize pertinent information for the project. Information that was recorded for injured, diseased, culled, and dead cows included identification numbers, freshening date, days in milk, lactation number, reason for removal or death given by farm, date of removal or death, and disease events with the date at which events occurred.

Eleven health EVENTS (e.g. categories) of interest were tracked throughout the period from enrollment until culling, death, or the study’s completion date. These events represented disease states commonly recorded industry-wide and on the participating dairies. Events of interest included 9 standalone categories: diarrhea, ketosis, lameness (hoof only), left displaced abomasum (LDA), mastitis, metritis, milk fever, pneumonia, and retained placenta. Two other events of interest, calving trauma and musculoskeletal injury (leg, hip, back), only were found as codes within culling or death (SOLD or DIED) events. Day-to-day oversight of health management including clinical diagnoses and on-farm computer database entries of disease, culling, and death were carried out by farm personnel. Input and training was provided by the dairy’s consulting veterinarian regarding clinical disease case definitions. Although case definitions and diagnoses undoubtedly varied to some degree between farms and personnel, this study was developed on the premise of using and comparing available disease data as-is.

A death certificate was created and a mortality code assigned to the DIED event for cows that died during the study [28]. This code included whether the cow was euthanized or died naturally, the location of death on the farm, a general management category such as ‘disease’ or ‘calving injury’, and underlying causative factors. Necropsies were not performed and underlying causes of death were based on farm employee or veterinarian assessments of health problems and previously recorded disease or injury events. Causes of death were ultimately attributed to one of the 11 disease states of interest where possible, or were classified as miscellaneous or unknown.

Culling of cows in this study was recorded as a SOLD event and attributed to either economic or biological reasons [4]. Cows classified as economic culls were removed based on an appraisal of profit versus loss due to milk production. Their removal from the herd was decided at the time by choice rather than dictated by the force of disease or injury. Biological culls were attributed to disease processes. The decision to remove the cow was considered mandatory to avoid ongoing welfare implications or death. Although most cows are culled from a herd due to a combination of underlying issues [4, 32], this study focused on the *best available* reason for removing the cow in light of the severity and urgency of ill health. Similar to causes of death, biological reasons for culling were ultimately attributed to one of the 11 disease states of interest where possible, or were listed as other miscellaneous disease processes.

### Disability weights

Disability weights are central to the comparable measurement of disease burden across diverse causes. A previous study was conducted using a survey of dairy health experts to formulate disability weights for the diseases of interest. Detailed methods related to the calculation of those weights are provided elsewhere [29]. Briefly, BetaPERT distributions were used to plot respondents’ opinions defined by minimum, most likely, and maximum values on a scale of 0 to 1 per disease state. A BetaPERT distribution fits a probability distribution around a respondent’s estimates to reflect uncertainty and variability in each response [33]. This was an applicable model to use for the respondents’ opinions as there was a certain level of variability in the estimated severity of diseases based on individual respondent’s personal experience. Individual distributions were combined to determine unique disability weights representing respondents’ collective opinion. These weights were grouped into classes to provide a calibration of the impact of similar conditions on dairy cows’ health. For a given disease the representative disability weight indicates the proportional impact on productive time. As an example, the disability weight for diarrhea is 0.4 representing an estimated 40% loss of productive time per day of clinical disease (Table 1).

**Table 1:** Disability weight class and estimated disease duration

DISEASE	Disability Weight Class	Duration (d)
Calving trauma	0.6	2
Diarrhea	0.4	2
Ketosis	0.5	2
Lame (hoof only)	0.5	5
LDA	0.6	3
Mastitis	0.5	5
Metritis	0.5	4
Milk Fever	0.5	1
Musculoskeletal injury*	0.6	5
Pneumonia	0.6	4
Retained Placenta	0.4	2

*leg, hip, back

### Unique cases and duration of disease

Every unique case of clinical disease was recorded for each cow throughout the 17 month enrollment period. No cows were enrolled with disease carried over from the dry-off period. A disease was considered unique and recorded as a new event for a given cow if it occurred 14 days or more from the termination of a previous, similar disease episode. This timeframe was determined based on recommended on-farm computer database protocols designed to identify new cases of disease as opposed to retreatment of the same disease episode [27].

The estimated duration of clinical disease is a key component of any summary measure of health that prioritizes both non-fatal health loss and death consistent with incidence-based DALY calculations [22]. For the diseases of interest in this study the average clinical durations were estimated based on professional opinion, farm personnel input, on-farm records, and multiple academic sources [34–41]. For each disease a range of possible durations exist, but for the purposes of this study a specific number of days were assigned (Table 1). This provided an estimated average duration of clinical impact per case even if the documented duration of treatment indicated otherwise.

### Calculation of the disease-adjusted lactation (DALact) summary measure

Similar to the human DALY, the DALact summary measure was derived from a comprehensive accounting of the time lost to disease and death. The DALact also accounted for the time lost due to forced (biological) removal. Whereas the DALY estimates the years of life lost, the DALact was formulated to estimate the days of milk lost relative to the average lactation length across the participating farms. Ultimately, the DALact accounted for days in milk (DIM) lost due to illness (DLI), forced removal (DLR), and death (DLD), relative to the average lactation length across the participating farms.

For the purposes of this study an incidence perspective was used to calculate the DLI to account for the estimated average durations of disease episodes [22]. The DLI was calculated as *I x DW x L* where *I* refers to the number of incident (i.e. newly diagnosed) cases for a given disease state, *DW* the disability weight, and *L* the average duration of a clinical disease problem. So that the DLI only accounted for days of illness on-farm and not after removal or death, *I* was based solely on the 9 standalone diseases of interest without input from culling or mortality records. In other words, no additional cases of standalone diseases were included based a cow’s designation when she left the farm, and no calving traumas or musculoskeletal injuries were included within the DLI at all.

The DLR and DLD were calculated as *N* x *L* where *N* represents the number of biological culls or deaths due to a cause, and *L* describes the days of life lost relative to an expected lactation length defined by an average calving interval. The participating dairies in the project had an average 13 month calving interval (390 days). Given a 60 day dry off period, this equated to an average of 330 days per lactation. The sum of the DALact calculations (DALact = DLI + DLR + DLD) ultimately described the overall burden of disease within a farm in terms of DIM lost as a function of the cumulative cases and severity of each disease for those lactating cows enrolled during the 17 month study period.

### Comparisons of frequency measures and the DALact

Diseases of interest were ranked based on the number of days lost on each dairy during the study period. These rankings were organized based on both the DLI and DALact. These measures of the burden of disease were compared against the ranking of 2 frequency measures: 1) morbidity-based cumulative disease frequency of standalone clinical disease events, and 2) removal-inclusive cumulative disease informed by culling and mortality data in addition to those standalone clinical disease events. Both cumulative frequency measures provided an accounting of unique incidents of disease per enrolled lactating cow during the 17 month study period. The morbidity-based cumulative measure of clinical disease was calculated based upon unique clinical episodes of the event of interest [e.g. MAST (mastitis), or LAME (hoof-related lameness), *without input from SOLD (culled) or DIED (mortality) records*], divided by the number of enrollments per dairy during the study period. This provided a proportional assessment of the number of cases of disease divided by the number of susceptible enrollees. This basic frequency measure accounting for cumulative disease is expected to be available on most U.S. dairies. The more robust, removal-inclusive measure of disease frequency included additional cases from culling and mortality records attributed to a given disease or injury, but without prior documentation of that particular health problem as a standalone health EVENT. Dependent upon the disease and the dairy, those records provided additional insight into the actual number of unique cases of a given disease.

## Results

A total of 5694 cows were enrolled during this 17 month study (Table 2). Of those, 1539 completed a lactation and were re-enrolled at the next freshening for a total of 7233 enrollments. Dairy 1 enrolled 1719 cows, of which 740 completed a lactation and were re-enrolled (2459 total enrollments). Dairy 2 enrolled 2135 cows, 398 of which were re-enrolled for a total enrollment of 2533. Dairy 3 enrolled 1840 cows, 401 of which were re-enrolled for a total enrollment of 2241. Dairy 1 culled 634 cows (26%) and had 160 cows die (7%). Dairy 1 had 319 economic culls versus 315 biological culls. Of the biological culls, 192 had a designation related to the 11 disease states of interest, and 123 were classified under miscellaneous diseases. Dairy 2 culled 590 cows (23%) and had 147 cows die (6%). Dairy 2 had 393 economic culls versus 197 biological culls of which 55 were classified as miscellaneous. Dairy 3 culled 598 cows (27%) and had 182 cows die (8%). Dairy 3 had 341 economic culls versus 257 biological culls of which 13 were classified as miscellaneous.

**Table 2:** Demographic data related to enrollments, culling, and death for each dairy

		Dairy 1	Dairy 2	Dairy 3
Enrolled	Initial enrollment	1719	2135	1840
	Re-enrolled	740	398	401
	Total	2459	2533	2241
Culled	Economic	319	393	341
	Biological	315	197	257
	Total	634	590	598
Died	Total	160	147	182

### Time lost to clinical phase of disease and injury (DLI)

The DLI are summarized in Table 3 for each dairy. Based on these calculations, mastitis and lameness were the predominant conditions affecting cow health on the participating dairies. Dairy 1 was most affected by mastitis with almost twice as much time lost to mastitis (2900 days) as to lameness (1535 days). In comparison, Dairy 2 had approximately a third more days lost to lameness (3025) as to mastitis (1958). Dairy 3 was about equally affected by lameness (2945 days) and mastitis (2563 days). Metritis was the third most costly disease in terms of time lost across all 3 dairies. The burden of other diseases varied by dairy. As can be seen in Table 3, none of the dairies used calving trauma or musculoskeletal injury as classifiers of active, clinical disease; rather, these events were utilized solely for culling or death.

**Table 3:** Number of diseased cows and clinical cases, and the days of life lost to illness (DLI)

	Dairy 1			Dairy 2			Dairy 3		
DISEASE	# Diseased	Cases	DLI^1^ (d)	# Diseased	Cases	DLI^1^ (d)	# Diseased	Cases	DLI^1^ (d)
Calving trauma	0	0	0	0	0	0	0	0	0
Diarrhea	65	67	54	122	136	109	45	45	36
Ketosis	128	128	128	107	107	107	7	7	7
Lame (hoof only)	481	614	1535	836	1210	3025	784	1178	2945
LDA	100	100	180	66	66	119	94	94	169
Mastitis	821	1160	2900	588	783	1958	674	1025	2563
Metritis	471	483	966	328	338	676	379	421	842
Milk Fever	50	50	25	22	22	11	40	40	20
Musculoskeletal injury*	0	0	0	0	0	0	0	0	0
Pneumonia	166	166	398	125	125	300	63	63	151
Retained Placenta	194	194	155	215	215	172	236	239	191

*leg, hip, back

^1^DLI = I x DW x L where I refers to the number of incident cases for a given disease state, DW the disability weight, and L the average duration of a clinical disease problem

### Time lost to forced (biological) removal (DLR), death (DLD) and overall DALact

The DLR, DLD, and DALact are presented in Table 4 for each dairy. Mastitis accounted for the most DLR across all 11 diseases of interest on the 3 participating dairies. Mastitis DLR ranged from 15,624 days on Dairy 2, to 18,857 days on Dairy 1, to 19,400 days on Dairy 3. The second highest DLR on Dairy 1 was due to pneumonia (8260 days), whereas lameness was second highest on Dairy 2 (4048 days), and musculoskeletal injuries came in second highest on Dairy 3 (13,727 days).

**Table 4:** The number of cows that were culled or died by designation and the average DIM at the time of culling or death

DISEASE (Dairy 1)	# Culled	Avg DIM cull	DLR^1^ (d)	# Died	Avg DIM died	DLD^1^ (d)	DALact (d)
Calving trauma	0	N/A	0	18	4	5868	5868
Diarrhea	1	42	288	7	60	1890	2232
Ketosis	0	N/A	0	3	17	939	1067
Lame (hoof only)	25	100	5750	5	39	1455	8740
LDA	14	68	3668	22	43	6314	10,162
Mastitis	109	157	18,857	8	111	1752	23,509
Metritis	0	N/A	0	4	13	1268	2234
Milk Fever	0	N/A	0	3	8	966	991
Musculoskeletal injury (leg, hip, back)	8	115	1720	16	78	4032	5752
Pneumonia	35	94	8260	9	66	2376	11,034
Retained Placenta	0	N/A	0	1	4	326	481
TOTAL categorized by disease above	192	129	38,543	96	47	27,186	72,070
Miscellaneous or unknown disease	123	120	25,830	64	89	15,424	N/A
Economic cull	319	205	N/A	N/A	N/A	N/A	N/A
DISEASE (Dairy 2)	# Culled	Avg DIM cull	DLR^1^ (d)	# Died	Avg DIM died	DLD^1^ (d)	DALact (d)
Calving trauma	0	N/A	0	17	2	5576	5576
Diarrhea	8	109	1768	4	112	872	2749
Ketosis	0	N/A	0	4	18	1248	1355
Lame (hoof only)	22	146	4048	7	75	1785	8858
LDA	10	40	2900	11	25	3355	6374
Mastitis	84	144	15,624	5	45	1425	19,007
Metritis	2	22	0	4	10	1280	1956
Milk Fever	0	N/A	0	2	3	654	665
Musculoskeletal injury (leg, hip, back)	4	75	1020	19	120	3990	5010
Pneumonia	12	128	2424	17	75	4335	7059
Retained Placenta	0	N/A	0	2	3	654	826
TOTAL categorized by disease above	142	130	27,784	92	56	25,174	59,435
Miscellaneous or unknown disease	55	152	9790	55	82	13,640	N/A
Economic cull	393	157	N/A	N/A	N/A	N/A	N/A
DISEASE (Dairy 3)	# Culled	Avg DIM cull	DLR^1^ (d)	# Died	Avg DIM died	DLD^1^ (d)	DALact (d)
Calving trauma	0	N/A	0	9	2	2952	2952
Diarrhea	0	N/A	0	11	81	2739	2775
Ketosis	0	N/A	0	4	10	1280	1287
Lame (hoof only)	79	169	12,719	10	127	2030	17,694
LDA	0	N/A	0	11	19	3421	3590
Mastitis	100	136	19,400	8	81	1992	23,955
Metritis	0	N/A	0	10	19	3110	3952
Milk Fever	0	N/A	0	2	5	650	670
Musculoskeletal injury (leg, hip, back)	53	71	13,727	24	87	5832	19,559
Pneumonia	12	158	2064	21	102	4788	7003
Retained Placenta	0	N/A	0	0	N/A	0	191
TOTAL categorized by disease above	244	134	47,910	110	68	28,794	83,628
Miscellaneous or unknown disease	13	135	2535	72	134	14,112	N/A
Economic cull	341	209	N/A	N/A	N/A	N/A	N/A

^1^DLR (days lost to removal) and DLD (Days lost to death) based on 13 mo calving interval minus a 60 day dry period.

Depending on the dairy, not all categories of disease were utilized to record biological culling and from 2 to 19% of biological culls were classified as miscellaneous. The 123 miscellaneous biological culls on Dairy 1 accounted for 25,830 days lost. This was over two-thirds (67%) as much time lost as the combined DLR (38,543 days) for the 192 biological culls attributed to the 11 diseases of interest on Dairy 1. In comparison, the combined DLR for disease-designated biological culls on Dairy 2 was 27,784 days, and on Dairy 3 it was 47,910 days. Contrast this to the miscellaneous biological culls on these dairies which accounted for a proportionally fewer 9790 days lost on Dairy 2, and 2535 days lost on Dairy 3.

The highest levels of DLD were attributed to LDA on Dairy 1 (6314 days), calving trauma on Dairy 2 (5576 days), and musculoskeletal injuries affecting the leg, hip, or back on Dairy 3 (5832 days). These 3 disease states along with pneumonia accounted for the 3 highest ranked causes of DLD for each of the participating dairies. As opposed to the classification of biological culls, all 11 diseases of interest were used to classify deaths and calculate the DLD. Nonetheless, there were numerous deaths ascribed to the miscellaneous or unknown disease category. This held true across all 3 dairies and the resultant levels of miscellaneous or unknown DLD amounted to approximately half as much time lost as the combined DLD for the other deaths. For example, Dairy 1 had 96 deaths categorized by disease that accounted for a combined DLD of 27,186 days. On the other hand, there were 64 deaths due to miscellaneous or unknown causes that accounted for 15,424 days lost. Dairy 2 had 92 deaths categorized by disease with a combined DLD of 25,174 days, versus 55 miscellaneous or unknown deaths with a DLD of 13, 640 days. Dairy 3 had 110 deaths categorized by disease with a combined DLD of 28,794 days, versus 72 miscellaneous or unknown deaths with a DLD of 14,112 days.

As a summary measure of health, the DALact consistently pointed to mastitis as the number one cause for concern on all 3 dairies (19,007 to 23,955 days). Secondary disease issues ranged from pneumonia (11,034 days), to lameness (8858 days), to musculoskeletal injuries (19,559 days). The DLI was outweighed by either the DLR or DLD for all diseases except retained placenta on Dairy 3, with a DLI of 192 days and DLR and DLD of 0 days. The combined time lost to the 3 most impactful diseases was in excess of 90 years per dairy and ranged from 96 years (34,924 days) on Dairy 2, to 168 years (61,208 days) on Dairy 3. Based on a 330 day lactation this ranged from 106 to 185 lost lactations. The total time lost due to the 11 diseases of interest amounted to 72,070 days (197 years; 218 lactations), 59,435 days (163 years; 180 lactations), and 83,628 days (229 years; 253 lactations) on Dairies 1, 2 and 3, respectively.

It should be noted that although miscellaneous or unknown diseases were not included in the DALact rankings due to their lack of specificity and no DLI estimates, as a generic category they tended to be larger than each of the individual disease categories. The combined days lost (DLR + DLD) to miscellaneous or unknown disease (Table 4) equated to 41,254 days on Dairy 1, and 23,430 days on Dairy 2. These values exceeded each of the individual disease DALact values on those dairies. Dairy 3 was presumably more systematic in disease recognition and classification and did not utilize the miscellaneous or unknown category as frequently. Consequently, the combined days lost to miscellaneous or unknown removal and death (16,647 days) fell below 3 of the specific disease DALact measures (mastitis: 23,955 days; musculoskeletal injury: 19,559 days; lame: 17,694 days).

### Frequency measures of disease and injury versus DLI and DALact estimates

On Dairy 1 there were 1160 distinctly identified events of mastitis equating to a 47% morbidity-based cumulative frequency (Table 5). An additional 59 cases (50% removal-inclusive cumulative frequency) were characterized from biological culls and deaths that were attributed to mastitis, without having had a previous record of that particular health problem. Although these additional cases are worthy of mention, the morbidity-based and removal-inclusive cumulative measures varied by 3% or less and were the same for most diseases across all 3 dairies.

**Table 5:** A comparison of disease cumulative frequency measures

DISEASE (Dairy 1)	Standalone dz. events	Morbidity-based cumulative frequency^a^	Removal-inclusive dz. events	Removal-inclusive cumulative frequency ^a^
Calving trauma	0	0%	18	1%
Diarrhea	67	3%	72	3%
Ketosis	128	5%	130	5%
Lame (hoof only)	614	25%	638	26%
LDA	100	4%	114	5%
Mastitis	1160	47%	1219	50%
Metritis	483	20%	485	20%
Milk Fever	50	2%	52	2%
Musculoskeletal injury (leg, hip, back)	0	0%	24	1%
Pneumonia	166	7%	184	7%
Retained Placenta	194	8%	195	8%
DISEASE (Dairy 2)	Standalone dz. events	Morbidity-based cumulative frequency ^b^	Removal-inclusive dz. events	Removal-inclusive cumulative frequency ^b^
Calving trauma	0	0%	17	1%
Diarrhea	136	5%	137	5%
Ketosis	107	4%	107	4%
Lame (hoof only)	1210	48%	1216	48%
LDA	66	3%	68	3%
Mastitis	783	31%	801	32%
Metritis	338	13%	339	13%
Milk Fever	22	1%	22	1%
Musculoskeletal injury (leg, hip, back)	0	0%	23	1%
Pneumonia	125	5%	131	5%
Retained Placenta	215	8%	217	9%
DISEASE (Dairy 3)	Standalone dz. events	Morbidity-based cumulative frequency ^c^	Removal-inclusive dz. events	Removal-inclusive cumulative frequency ^c^
Calving trauma	0	0%	9	0%
Diarrhea	45	2%	47	2%
Ketosis	7	0%	9	0%
Lame (hoof only)	1178	53%	1205	54%
LDA	94	4%	95	4%
Mastitis	1025	46%	1047	47%
Metritis	421	19%	423	19%
Milk Fever	40	2%	41	2%
Musculoskeletal injury (leg, hip, back)	0	0%	77	3%
Pneumonia	63	3%	76	3%
Retained Placenta	239	11%	239	11%

^a^Cumulative frequency based on the number of standalone or removal-inclusive (biological culling and death) disease events divided by 2459 enrollments

^b^Cumulative frequency based on the number of standalone or removal-inclusive (biological culling and death) disease events divided by 2533 enrollments

^c^Cumulative frequency based on the number of standalone or removal-inclusive (biological culling and death) disease events divided by 2241 enrollments.

Regardless of the cumulative measure used, mastitis and lameness were the 2 most commonly occurring diseases of the 11 diseases of interest (Table 6). Metritis was the third most common disease, and retained placenta the fourth across all 3 dairies. All other disease states had < 10% cumulative frequency with calving trauma, diarrhea, LDA, milk fever and musculoskeletal injury consistently at ≤5% (Table 5).

**Table 6:** Disease-adjusted lactation (DALact) days of life lost compared against morbidity-based and removal-inclusive (biological culling and death) cumulative frequency rankings of diseases, and the ranking of time lost to on-farm clinical disease alone (DLI). A rank of 1 indicates the highest cumulative frequency or most time lost versus a rank of 11 indicating the lowest cumulative frequency or least time lost

DISEASE (Dairy 1)	Morbidity-based cumulative frequency rank	Removal-inclusive cumulative frequency rank	DLI rank	DALact rank
Calving trauma	10	11	10	5
Diarrhea	8	8	8	8
Ketosis	6	6	7	9
Lame (hoof only)	2	2	2	4
LDA	7	7	5	3
Mastitis	1	1	1	1
Metritis	3	3	3	7
Milk Fever	9	9	9	10
Musculoskeletal injury (leg, hip, back)	10	10	10	6
Pneumonia	5	5	4	2
Retained Placenta	4	4	6	11
DISEASE (Dairy 2)	Morbidity-based cumulative frequency rank	Removal-inclusive cumulative frequency rank	DLI rank	DALact rank
Calving trauma	10	11	10	5
Diarrhea	5	5	7	7
Ketosis	7	7	8	9
Lame (hoof only)	1	1	1	2
LDA	8	8	6	4
Mastitis	2	2	2	1
Metritis	3	3	3	8
Milk Fever	9	10	9	11
Musculoskeletal injury (leg, hip, back)	10	9	10	6
Pneumonia	6	6	4	3
Retained Placenta	4	4	5	10
DISEASE (Dairy 3)	Morbidity-based cumulative frequency rank	Removal-inclusive cumulative frequency rank	DLI rank	DALact rank
Calving trauma	10	10	10	7
Diarrhea	7	8	7	8
Ketosis	9	10	9	9
Lame (hoof only)	1	1	1	3
LDA	5	5	5	6
Mastitis	2	2	2	1
Metritis	3	3	3	5
Milk Fever	8	9	8	10
Musculoskeletal injury (leg, hip, back)	10	6	10	2
Pneumonia	6	7	6	4
Retained Placenta	4	4	4	11

On all dairies the 3 highest ranked diseases were ranked the same by both cumulative measures and the DLI (Table 6). Dairy 1 and 2 had some minor discrepancies in cumulative frequency versus DLI for those diseases ranked from fourth to eleventh by any measure. For example, on Dairy 1 retained placenta was ranked fourth by both cumulative measures (8%) and sixth via DLI (155 days). Dairy 3 provided a somewhat different perspective in that both cumulative measures and DLI were ranked the same for the 5 highest ranked disease events of interest. The 5 lowest ranked diseases also were ranked the same by morbidity-based cumulative frequency and DLI measures. However, the inclusion of biological culling and mortality data increased the removal-inclusive cumulative frequency of musculoskeletal injuries from 0% to 3%. That moved its removal-inclusive cumulative frequency ranking to sixth and displaced the remaining health events downward.

Contrast the results above to a comparison of cumulative frequency rankings versus the DALact ranking shown in Table 6. On Dairy 1, only 2 of the rankings were the same by all measures with mastitis ranked first (47% or 50% cumulative frequency; 23,509 days), and diarrhea ranked eighth (3% cumulative frequency; 2232 days). The second ranked disease by cumulative frequency was lameness (25% or 26%) which was ranked fourth by the DALact (8740 days). Conversely, the second ranked disease by the DALact was pneumonia (11,034 days) which was ranked fifth (7%) by cumulative frequency. Of note, by assessing the time-based burden of disease the DALact provided a robust accounting of health events otherwise overlooked by frequency measures. In this case, calving trauma and musculoskeletal injuries were ranked fifth (5868 days) and sixth (5752 days) by the DALact but were at the bottom of the scale by either measure of cumulative disease (0% or 1%).

Dairy 2 also saw fluctuations in the rankings between measures of cumulative frequency and DALact. The 3 highest ranked cumulative measures (lameness: 48%; mastitis: 31% or 32%; metritis: 13%) were ranked second (8858 days), first (19,007 days), and eighth (1956 days), respectively, by the DALact. The third highest DALact (pneumonia: 7059 days) was ranked sixth (5%) by cumulative frequency. Similar to Dairy 1, calving trauma and musculoskeletal injuries were ranked a notable fifth (5576 days) and sixth (5010 days) by the DALact but were near the bottom of the rankings by cumulative frequency (0% or 1%).

Dairy 3 had differences in rankings comparable to the other dairies. The 3 highest ranked cumulative frequency measures were ranked the same as Dairy 2 (lameness: 53% or 54%; mastitis: 46% or 47%; metritis: 19%) but were ranked third (17,694 days), first (23,955 days), and fifth (3952 days), respectively, by the DALact. However, on this dairy musculoskeletal injuries were ranked second per the DALact (19,559 days) and sixth by the removal-inclusive cumulative measure (3%), but remained tied for last with calving trauma per the morbidity-based cumulative measure (0%).

## Discussion

There is an increasing push for dairy production to be scientifically grounded and ethically responsible in the oversight of animal health and well-being. Addressing underlying challenges affecting the quality and length of productive life necessitates novel assessment and accountability metrics. Such metrics can provide a structured and transparent measurement of farm animal care and welfare, and improve governance and oversight of production practices and health outcomes [6]. We propose a disease-adjusted metric (DALact) to help evaluate dairy cow health and well-being. The DALact provides a time-based method for addressing the complementary nature of disease and death and optimizing health care management on dairies.

To calculate the DALact we relied on an incidence perspective rather than the simplified prevalence-based approach currently used to calculate the human DALY [22, 23]. A prevalence perspective helps overcome challenges related to accounting for lifetime disease events including congenital and chronic conditions singularly recorded at the time of diagnosis. However, it shifts a greater burden of disease onto deaths as compared to non-fatal health loss due to the removal of disease duration from the equation. Ultimately, calculating the DLI using an incidence perspective aligned more closely with our interest in comprehensively accounting for the burden of disease during lactations rather than across lifetimes, using standard records documenting each unique episode of common diseases in tandem with estimates of average durations. That said, we focused on the cumulative frequency of incident cases rather than an incidence measure per se (i.e. no prescribed period of time was attached to the frequency measures). This provided an uncomplicated count of unique disease episodes expected to be available for comparative analysis on most U.S. dairies with or without input from culling and mortality records.

In the end, the DALact combined disability weights, estimated average durations of disease, average lactation length, and the cumulative frequency of disease. The results from this study were based on point estimate classifications of disability weight distributions [29], durations of disease based on multiple inputs, and farm specific calving intervals. Using an estimated average duration per case, regardless of whether the documented duration of treatment indicated otherwise, provided a means for standardizing durations of diseases that tend to be recorded based on treatments rather than clinical longevity per se. Situations that allow for more direct individual oversight and recording of clinical durations might benefit from incorporating case-by-case assessments of disease duration rather than relying on averages.

Although variations in calving intervals and consequent lactation lengths are dependent upon many aspects of farm management, the salient point is that the calculation of burden of disease is reliant on a standardized expectation of longevity. This study attempted to provide a simple but comprehensive estimate of the disease burden based on the average completed lactation length for the herd. The purpose was to identify and focus on the spectrum of health problems in total by viewing disease states across all ages and lactations equally. There is room for flexibility here, though, in that a more conservative estimate of time lost might be based on average lactation lengths inclusive of economic removals (e.g. without a requisite calving interval) or scaled in terms of age or lactation number. This would acknowledge that a lactation length based on calving intervals is dictated by “survivors” and that the time lost in later lactations is progressively more likely and less costly to the system as a whole.

Differences in the frequency of disease outcomes within this study may have been due as much to non-sampling error as to the actual cumulative frequency. Sources of non-sampling error include selection bias in the population studied, data collection issues, variations in case definitions, and the use of different diagnostics technologies [18]. Diagnostic outcomes undoubtedly were influenced by employee training, experience, adherence to protocols, and timing within the progression of disease. For example, although it is possible that Dairy 3 had substantially fewer cases of ketosis relative to the other dairies, it is plausible that ketosis simply was not diagnosed or recorded similarly. Furthermore, this study followed set protocols that considered a disease unique and recorded it as a new event for a given cow if it occurred 14 days or more from the termination of a previous, similar disease episode. Although there may have been cases for which this actually identified a chronic disease state rather than a unique episode, it nonetheless accounted for the duration of disease in line with an incidence perspective. Ultimately this study was dependent upon using the dairies’ records as-is to provide a comparative assessment of health problems based on realistic, available data with an emphasis on reasons for culling and death.

In this study there were unequal risk periods from freshening to disease, removal, or death based on enrollment timing relative to the study’s completion. Although dimensionless, the cumulative frequencies were influenced by the length of a given lactation. Therefore, the frequency of early lactation diseases such as calving trauma, metritis, and retained placenta were inflated relative to other, more equally distributed health issues such as lameness. For example, a cow that was enrolled on March 1st, 2014 and calved again on April 1st, 2015 was exposed to a full 13 month calving interval for disease manifestation. However, she then had just under 2 months from the time of her second enrollment until the completion date of the study, May 26th, 2015, during which time certain early lactation health problems were more likely than others. Although this study design focused attention on early lactation disease and injury, it affected both cumulative and DALact measures equally without compromising the study’s focus on a comparative analysis of the impact of disease.

Evidence suggests that farmers are increasingly constrained from making economic culling decisions due to increased disease-related reasons such as lameness or injury [7]. For the purposes of this study biological culling was assigned to those cows that required urgent removal from the herd to avoid welfare implications or death. This provided a more conservative estimate of forced removals than would have been given by broadly defining biological culls as those animals without a productive future [4]. This limited the impact of biological culling on the overall assessment of the disease burden to those instances with distinctly aligned health problems. Even so, the total DLR outweighed the DLI and DLD for the aggregate 11 diseases of interest on all three dairies. An argument could be made for a less conservative approach acknowledging that most removals, including miscellaneous biological and economic culls, have underlying biological associations that could be accounted for in a disease-adjusted summary measure of health. The limitation to such an approach is that dairy record systems tend to focus on specific, treatable diseases with limited and inconsistent information regarding culling and death [9, 25]. This project focused on biological culls with documented underlying disease or injury at the time of removal. Given better health records, future derivatives of a dairy summary measure of health might more thoroughly explore the impact of subclinical and cumulative disease processes on removals as a whole.

Dairy health records often lack the accuracy and consistency needed to be useful for informing herd-level management decisions [27]. Using miscellaneous biological culls as a catch-all classification for poorly attributed ill-health limited the accuracy and value of the assessment of the burden of disease. This was true as well for those deaths that were categorized as miscellaneous or unknown. By quantifying these losses in terms of DIM a measure of diminished information was documented for these generic records of removal and mortality. For Dairy 1 this amounted to a total of 41,254 days lost to miscellaneous or unknown disease associated with culling or death. That was 57% as much time as that attributed to the full DALact for the 11 disease states of interest (72,070 days). Even though proportionally fewer days were lost to removals and death from miscellaneous or unknown disease on the other dairies, those generic losses still amounted to 39% (23,430 days/59,435 days; Dairy 2) and 20% (16,647 days/83,628 days; Dairy 3) as much time as that attributed to the combined DALact for the diseases of interest. This loss of detail ultimately impacted the dairies differently depending on their use of disease categories, but collectively highlighted the benefit in health information and oversight that might be gained from improved removal and mortality records [7, 9].

Regardless of the information loss from miscellaneous and unknown classifications, the DALact’s temporal-basis and incorporation of those available data related to specific causes of culling and death provided a depth of analysis surpassing that of disease frequency measures. A morbidity-based assessment of cumulative frequency indicated that the predominant health issue on Dairy 1 was mastitis (47%), with lameness (25%) and metritis (20%) second and third in terms of importance, respectively. This held true as well with a more thorough, removal-inclusive assessment of cumulative frequency that incorporated culling and mortality records. This also was in line with the DLI rankings based solely on the time lost to standalone clinical disease. Based on these accountings of disease, a dairy’s health managers likely would focus their energies and resources on combating mastitis with additional efforts directed toward reducing lameness and metritis issues. Alternatively, if health decision-making and planning processes included an assessment of lost productive time inclusive of culling and death, this dairy’s health managers would remain focused on mastitis (23,509 days) but might direct successive efforts toward reducing the less frequent but substantially more time-abolishing problems of pneumonia (11,034 days) and LDA (10,162 days).

On Dairy 2 a similar pattern might play out with the primary focus shifting from lameness (48% cumulative frequency; 8858 days) to mastitis (19,007 days; 31% or 32% cumulative frequency), and additional efforts directed toward mitigating both lameness and pneumonia (7059 days). Dairy 3 presented a particularly interesting comparison in that even though lameness (53% or 54% cumulative frequency; 17,694 days) and mastitis (46% or 47% cumulative frequency; 23,955 days) remained near the top of the list for all measures, musculoskeletal injuries rose from the bottom of morbidity-based cumulative and DLI measures (0%; 0 days), to sixth via a removal-inclusive cumulative measure (3%), and was identified as the second most important health issue on the farm based on the DALact (19,559 days). On all 3 dairies the inclusion of time-based culling and mortality statistics lifted both musculoskeletal injuries and calving trauma from effectively irrelevant (0% morbidity-based cumulative frequency; 0% to 3% removal-inclusive cumulative frequency) to areas of concern accounting for appreciable time lost (2952 to 19,559 days). Presumably a producer presented with this information would investigate the underlying causes of traumatic injuries and redirect resources to minimize their occurrence and effects.

It is important to emphasize the point that a summary measure of dairy health goes beyond simply linking morbidity to culling and mortality in a standardized fashion. A summary measure speaks to the burden of disease on both the well-being and productivity of individuals and populations. When framed as lost days, years, or lactations the various health issues on a farm are more comprehensible than they may be by frequency measures alone. Consider the findings from this study that identified mastitis as a primary health concern across farms. Cumulative measures ranged from 31% to 50%, numbers that can be difficult to conceptualize for dairy health care managers. On the other hand, using the DALact metric the impact of mastitis can be spoken of in terms of the 58 to 73 lost lactations per farm. Such an alternative accounting of disease highlights the lost opportunity costs of production as well as the burden of mastitis on life as a whole.

Increases in levels of mortality are widespread in modern dairy production systems and novel control schemes are needed to reduce the economic and animal welfare costs, as well as the reputational risk posed to the industry. Furthermore, the timing and fates of animals that exit dairy farms through culling or death are informative in their reflection of management conditions and production efficiencies [7]. Frequency measures of disease focus attention on the occurrence of standalone clinical disease, without necessarily accounting for the overall burden of disease on affected animal populations. Although lost milk production and the cost of disease management often serve as a proxies, they do not provide a standardized, comparative accounting of the burden of disease over time, populations, disease states, and outcomes such as culling and death. Ideally, both frequency and DALact measures of disease would be viewed in tandem to address the composite underlying problems affecting dairy cow health and well-being. The DALact is, after all, an extension of disease-related data and for any given dairy this would increase the utility and value of accurate and consistent data collection related to clinical health problems, and highlight the importance of record designations focused on underlying causes of biological culling and death.

Although this initial study focused on the burden of disease during lactation, the concept ultimately should be applied across a cow’s life in an effort to determine the most important contributors to lifetime burdens of disease and consequent opportunity costs. Yet many of the same challenges that complicate human medical epidemiology and the calculation of lifetime DALYs influence the accuracy and consistency of dairy health records as well. Consistency in clinical assessments, diagnostic test frequency and accuracy, estimations of disease duration and repeat episodes, age-related factors, and comorbidities all impact the documentation of disease [22, 25]. Furthermore, whereas human medical epidemiology has historically focused on mortality within health evaluations, dairy record systems have historically focused on non-fatal health problems. Underlying causes of culling and death are rarely codified or recorded to a level that allows for meaningful decision making [28]. These issues create challenges for incorporating a summary measure of lifetime health into routine assessments of dairy cow health and well-being. Future endeavors undoubtedly can benefit from considering methodological revisions in accordance with the DALY’s modifications of disability weights to account for various sequelae including culling and death, adjustments for comorbidity, temporality, and time discounting, and use of prevalence versus incidence perspectives [18, 22]. Taking these considerations into account, a lifetime-based summary measure of dairy cow health should provide an appreciation of the big picture, a comparison of both acute and chronic diseases, injuries, and risk factors, and an understanding of the most important contributors to health loss in a given time, place, and age group [23].

## Conclusions

The results from this study suggest that a time-based summary measure of dairy cow health 1) provides an enhanced assessment of clinical disease frequency measures, and 2) necessitates improved records across the continuum of morbidity, culling, and mortality. Herd health managers should work to improve dairy records such that all occurrences, not just treated occurrences, are entered for disease events of interest. Furthermore, culling and mortality records should capture relevant causality whenever possible. Current dairy records may not fully support a comprehensive accounting of the burden of disease based on the DALact metric. Nonetheless, available data related to disease, culling, and death can be incorporated into the DALact calculation to aid animal health oversight. Modest improvements to standard records would allow a summary measure of dairy cattle health to be reliably included within evaluations that guide management decisions, and direct resources toward areas that most impact the health and welfare of a herd.

## Abbreviations

DALact: Disease-adjusted lactation days of life lost
DLD: Days of life lost due to death
DLI: Days of life lost due to illness
DLR: Days of life lost due to forced removal (biological culling)
